# Fufang Taizishen Granules Attenuate Chemotherapy‐Induced Intestinal Mucositis by Modulating Gut Microbiota and Amino Acid Metabolism

**DOI:** 10.1002/fsn3.70789

**Published:** 2025-08-11

**Authors:** Yongjun Kan, Yingying Liu, Li Zhao, Chang Jiang, Wensheng Pang, Bianhong Zhang, Wenxiong Lin, Juan Hu

**Affiliations:** ^1^ Fujian Academy of Chinese Medical Sciences Fuzhou P.R. China; ^2^ The Second Affiliated Hospital of Fujian University of Traditional Chinese Medicine Fuzhou P.R. China; ^3^ Fujian University of Traditional Chinese Medicine Fuzhou P.R. China; ^4^ Fujian Key Laboratory for Agroecological Processes and Safety Monitoring Fujian Agriculture and Forestry University Fuzhou P.R. China

**Keywords:** amino acid metabolism, Fufang Taizishen granules, gut microbiota, intestinal mucositis

## Abstract

Chemotherapy‐induced intestinal mucositis is a severe adverse effect affecting cancer patients, and there are currently no effective strategies for its prevention. Fufang Taizishen granules (FFTZS), a traditional Chinese medicine (TCM) known for its anti‐inflammatory, anti‐fatigue, and immunostimulatory properties, have shown therapeutic potential. However, the effects of FFTZS on chemotherapy‐induced intestinal mucositis and its underlying mechanisms remain unclear. A mouse model of intestinal mucositis was induced using 5‐FU and irinotecan to assess the protective effects of FFTZS. The study found that FFTZS enhanced food intake, reduced diarrhea index, and improved histopathological damage in colonic tissues. FFTZS also reduced inflammatory cell infiltration and inhibited apoptosis. Moreover, FFTZS treatment increased the expression of Claudin‐1 and ZO‐1 proteins. Microbial analysis revealed an enrichment of beneficial bacteria and a reduction of opportunistic pathogens, whereas metabolomic analysis showed that FFTZS corrected amino acid metabolic disturbances induced by chemotherapy. These findings suggest that FFTZS mitigates chemotherapy‐induced intestinal mucositis by preserving mucosal integrity, modulating gut microbiota, and restoring metabolic homeostasis.

## Graphical abstract

Fufang Taizishen granules (FFTZS) alleviate chemotherapy‐induced intestinal mucositis by enhancing mucosal integrity, reducing inflammation, and modulating gut microbiota composition. In a mouse model, FFTZS improved clinical symptoms, restored tight junction proteins, and corrected amino acid metabolic disturbances. These findings highlight FFTZS as a promising therapeutic strategy for managing mucositis during chemotherapy.

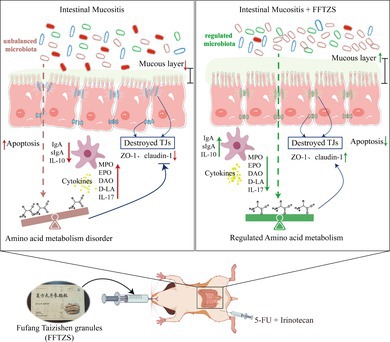

## 1 Introduction

Chemotherapy‐induced intestinal mucositis is a severe adverse reaction experienced by cancer patients undergoing treatment, characterized by painful inflammation and ulceration of the gastrointestinal mucosa (He et al. 2023). This condition not only leads to symptoms such as anorexia, vomiting, and diarrhea but also frequently results in treatment delays or cessation. Epidemiological studies reveal that approximately 40% of patients receiving standard‐dose chemotherapy develop mucositis, manifesting as pain, inflammation, diarrhea, weight loss, and infection. Furthermore, nearly 100% of patients undergoing high‐dose chemotherapy experience mucositis (Dahlgren and Lennernäs 2023). Notably, commonly used chemotherapeutic agents, such as 5‐fluorouracil (5‐FU) and irinotecan, induce severe mucositis (Alcorta et al. 2024). Beyond causing debilitating symptoms, chemotherapy‐induced mucositis substantially diminishes patients' quality of life, often necessitating dose reductions that impede chemotherapy progression, increase hospitalization needs, and potentially heighten mortality risk. Currently, there is no definitive cure for intestinal mucositis, with clinical management limited to pain relief and the use of loperamide to control diarrhea (Bai, Zhao, et al. 2024; Xu et al. 2024). Given these limitations, the urgent need for effective therapies targeting mucositis is evident.

The intestine is a multifunctional organ responsible for food digestion, nutrient absorption, and serving as a critical defense barrier to regulate the intestinal environment and resist harmful pathogens such as bacteria, toxins, and viruses (Vancamelbeke and Vermeire 2017). The intestinal mucosal barrier system comprises intestinal epithelial cells, tight junction proteins between adjacent epithelial cells, and a mucus layer covering the epithelium. This system plays a crucial role in preventing harmful substances, including bacteria and endotoxins, from translocating across the mucosa into the bloodstream. Tight junction proteins, which include transmembrane and perimembranous protein families, are essential for maintaining the intestinal mucosal barrier's functionality and permeability. The mucus layer forms a key defensive barrier against pathogenic microbial invasion, and its disruption can trigger intestinal microbial infections of epithelial cells, leading to mucositis (Desai et al. 2016). Studies have shown that treatment with 5‐FU and irinotecan often results in significant damage to the intestinal mucosal barrier. Moreover, the gut microbiota plays a vital role in maintaining intestinal homeostasis and integrity. Evidence from murine models and cancer patients indicates that targeting the gut microbiota can alleviate intestinal mucositis and enhance the efficacy of anticancer drugs (Cheng et al. 2020).

Recently, TCM has garnered increasing attention for its potential benefits in managing post‐chemotherapy side effects due to its relatively low toxicity and side effects. Fufang Taizishen granules (FFTZS), a traditional Chinese prescription, comprise *Pseudostellaria heterophylla*, *Ganoderma lucidum*, *Poria cocos*, *Crataegus pinnatifida* , *Hordei Fructus Germinatus* and *Oryzae Fructus Germinatus*, zinc sulfate, calcium gluconate, and ferric ammonium citrate. In TCM, FFTZS is believed to possess “Qi‐replenishing,” “fluid‐generating,” and “spleen‐invigorating” properties.

Given the anti‐inflammatory, anti‐fatigue, and immune‐stimulating properties of FFTZS's active ingredients, we hypothesized that FFTZS may exert beneficial effects on intestinal health. This study aimed to evaluate and elucidate the protective effects of FFTZS on chemotherapy‐induced intestinal mucosal damage in mice treated with 5‐FU and irinotecan. We focused on its impact on intestinal barrier function, gut microbiota composition, and microbial metabolites. Additionally, we sought to determine whether these effects could be attributed to FFTZS‐mediated regulation of microbial metabolic pathways, providing experimental evidence for its potential clinical application.

## 2 Materials and Methods

### 2.1 Animals and Experimental Design

Specific‐pathogen‐free grade male BALB/c mice (8 weeks old, weighing ~25 g) were purchased from Shanghai SLAC Laboratory Animal Co. Ltd. The mice were housed under a 12‐h light/dark cycle at 24°C ± 2°C and 55% ± 10% relative humidity, with free access to food and water. All animal experiments were conducted following the institutional guidelines for animal care approved by the Ethics Committee of Experimental Animals at Fujian Academy of Chinese Medical Sciences (approval number: FJATCM‐IAEC2021007). Mice were randomly assigned to experimental groups using a random number table. Investigators responsible for sample analysis, including histopathology, immunostaining, and biochemical assays, were blinded to group allocations to minimize potential bias.

After a 1‐week acclimatization period, the mice were randomly divided into five groups (*n* = 8 per group):

- Control group (CON): Mice received 0.9% saline (5 mL/kg) via oral gavage.
- FFTZS control group (FF‐C): Mice received FFTZS solution (1 g/kg/day) via oral gavage.
- Model group (MOD): Mice received intraperitoneal injections of 5‐FU (25 mg/kg) and irinotecan (25 mg/kg) from Day 8 to Day 11.
- Early intervention group (EI): Mice received FFTZS solution (2.5 g/kg) starting 1 week before 5‐FU and irinotecan administration, continuing for 18 days.
- Late intervention group (LI): Mice received FFTZS solution (2.5 g/kg) starting immediately after the first administration of 5‐FU and irinotecan, with FFTZS administered 1 h post‐chemotherapy.

### 2.2 General Health Monitoring

Food consumption was calculated based on the difference between the provided food quantity and remaining food. Weight loss was determined as the difference in body weight between Day 15 and Day 18. The Disease Activity Index was assessed using weight loss, stool consistency, and stool bleeding scores as described previously (Ma et al. 2021).

### 2.3 Histological Evaluation

Colonic tissues were fixed in 4% paraformaldehyde, embedded in paraffin, and sectioned. Hematoxylin and eosin staining was performed to evaluate morphological changes in the colon. The villus height and crypt depth were measured to assess tissue structural alterations. The villus‐to‐crypt ratio was calculated to evaluate the absorptive capacity. Measurements were performed by an independent pathologist blinded to the treatment groups.

### 2.4 Alcian Blue Staining

Paraffin sections were deparaffinized and hydrated, followed by staining with Alcian blue solution for 15 min. Slides were gently rinsed, counterstained with nuclear fast red for 3 min, and dehydrated using absolute ethanol and xylene. Finally, the sections were mounted with neutral gum.

### 2.5 Periodic Acid‐Schiff (PAS) Staining

Colon tissues were fixed using Carnoy's Fixative Solution, embedded in paraffin, and sectioned. After deparaffinization, PAS staining was performed using a PAS staining kit (Servicebio, Wuhan, China). Sections were then dehydrated in ethanol and xylene before mounting with neutral gum.

### 2.6 TUNEL Assay

Colonic tissue was fixed in 4% paraformaldehyde, embedded in paraffin, and sectioned at 4 μm thickness. TUNEL staining was performed using a TUNEL assay kit following the manufacturer's instructions. Images from five random fields per section were captured to evaluate apoptosis levels in colonic tissues.

### 2.7 Immunohistochemistry

Paraffin‐embedded colonic sections underwent deparaffinization, antigen retrieval, and blocking. Sections were incubated with primary antibodies against ZO‐1 or Claudin‐1 (1:200) overnight. Following PBS washes, DAB staining was performed using an immunohistochemistry kit. Five random fields per sample were imaged, and positive expression (brown staining) was quantified using ImageJ software.

### 2.8 Enzyme‐Linked Immunosorbent Assay

Total protein from colonic tissues was extracted using cold RIPA lysis buffer, and concentrations were determined using a BCA assay kit. Enzyme‐linked immunosorbent assay kits were employed to quantify MPO, EPO, IgA, and sIgA in colonic tissues and DAO, D‐LA, IL‐10, and IL‐17 in serum following the manufacturer's instructions.

### 2.9 Microbial DNA Extraction and 16S rRNA Sequencing

Gut microbial DNA was extracted from colonic fecal contents using HiPure Soil DNA Kits (Magen, Guangzhou, China) per the manufacturer's protocol. The V3–V4 region of the 16S rRNA gene was amplified using universal primers (338F: 5′‐ACTCCTACGGGAGGCAGCA‐3′, 806R: 5′‐GGACTACHVGGGTWTCTAAT‐3′). Libraries were prepared, and amplicons were sequenced on an Illumina HiSeq 2500 platform (Beijing Biomarker Technologies Co. Ltd.).

### 2.10 Quantification of *Akkermansia* and *Roseburia*

Total genomic DNA was extracted from feces using the fecal genomic DNA extraction kit (Magen) according to the manufacturer's instructions. Total bacterial DNA was quantified by qRT‐PCR, with normalization to universal 16S rRNA gene levels, and the relative abundances of *Akkermansia* and *Roseburia* were calculated accordingly. Primer sequences are listed in Table S1.

### 2.11 Metabolomics Analysis

Freeze‐dried colonic fecal samples (50 mg) were homogenized in 1.5 mL EP tubes with 1000 μL extraction solvent (methanol–acetonitrile–water = 2:2:1, v/v, containing 20 mg/L internal standard). Samples were vortexed, sonicated in an ice bath, and centrifuged (4°C, 12,000 × *g*, 15 min). The supernatants were dried under vacuum, reconstituted in 160 μL acetonitrile–water (1:1, v/v), sonicated again, and centrifuged for UPLC‐HRMS analysis. The conditions for mass spectrometry and chromatography, as well as the metabolite identification procedures, are described in detail in Section S2.

### 2.12 Data Analysis

Data were expressed as mean ± standard deviation (SD). For comparisons involving multiple groups, one‐way ANOVA followed by Tukey's post hoc test was used. In multidimensional analyses involving large datasets (e.g., microbiota and metabolomics), false discovery rate correction was applied using the Benjamini–Hochberg method to control for multiple testing. Adjusted *q*‐values < 0.05 were considered statistically significant. Sequencing data were processed using QIIME, and taxonomic annotations were performed using the NCBI database. Normalization was based on the sample with the lowest sequence count. Bioinformatics analyses were conducted on the Biomarker BioCloud platform (www.biocloud.net).

## 3 Results

### 3.1 FFTZS Increases Food Intake and Alleviates Diarrhea in Chemotherapy‐Treated Mice

**FIGURE 1:**
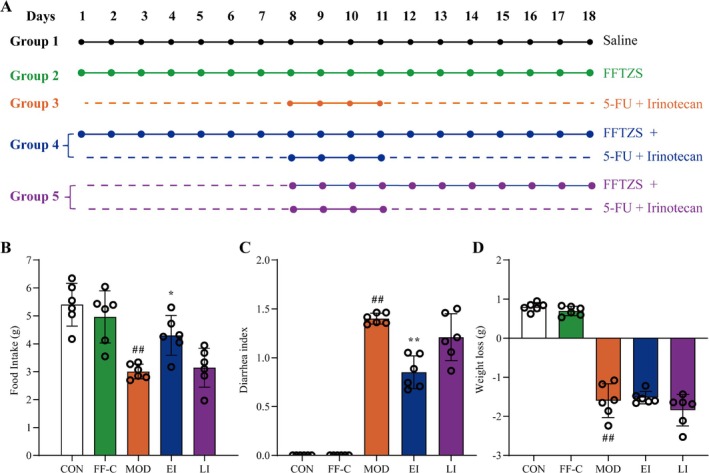
Schematic representation of the experimental design (A) and changes in food intake (B), diarrhea index (C), and weight loss (D) of mice in different treatment groups.

To assess the effects of FFTZS on chemotherapy‐induced adverse symptoms, we examined food intake, body weight, and diarrhea index (Figure 1). Compared with the CON group, the MOD group displayed significant chemotherapy‐associated adverse effects, including reduced food intake, weight loss, and pronounced diarrhea. Notably, the EI group showed a significant increase in food intake (*p* < 0.05) and a reduced diarrhea index compared with the MOD group. However, no significant improvement in weight loss was observed in the EI or LI groups compared with the MOD group. These results suggest that FFTZS intervention mitigates chemotherapy‐induced anorexia and alleviates diarrhea but does not significantly affect weight loss.

### 3.2 FFTZS Alleviates Pathological Damage in Colonic Tissues of Chemotherapy‐Treated Mice

**FIGURE 2:**
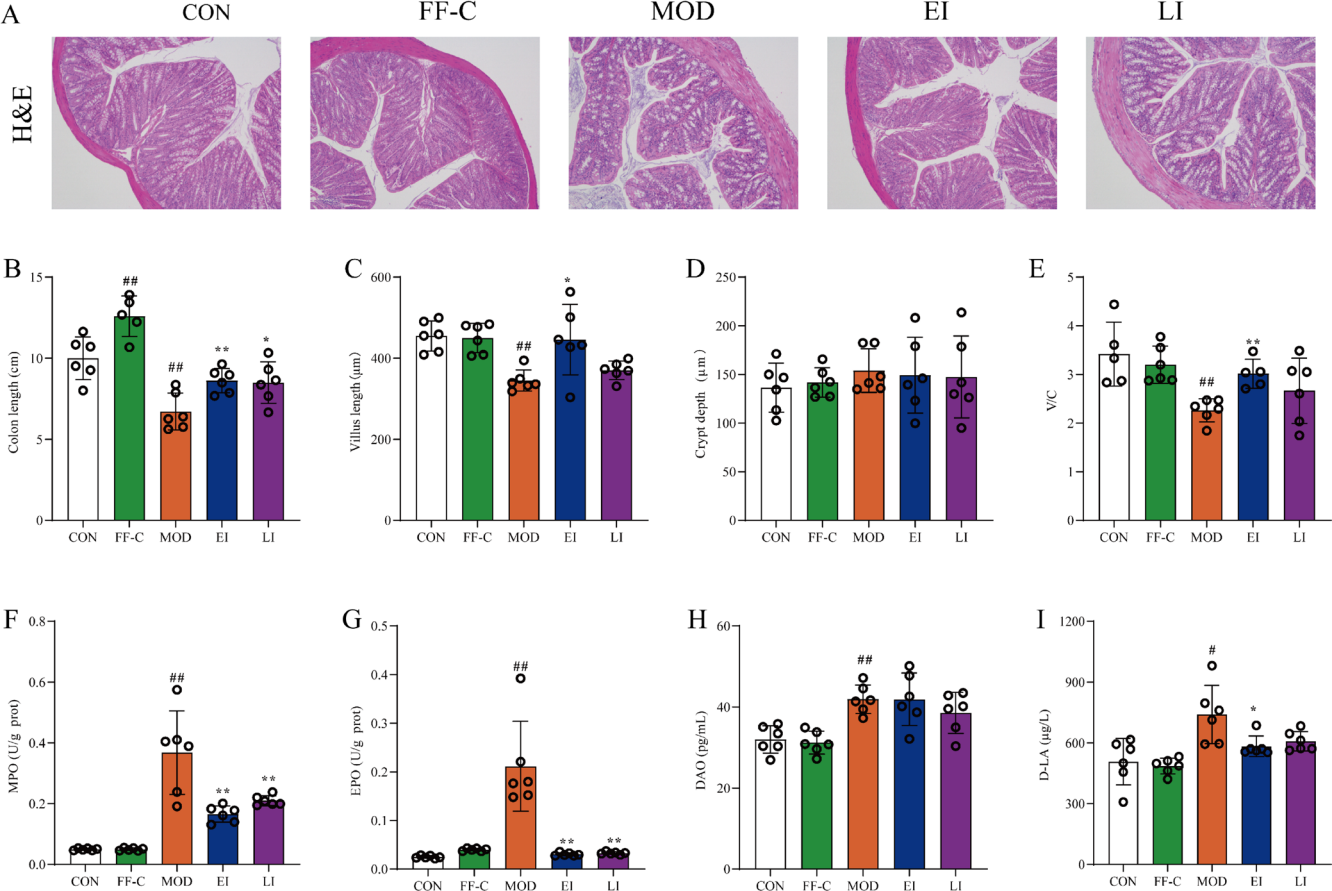
Effects of FFTZS on colonic tissue morphology and inflammatory infiltration in chemotherapy‐treated mice. (A) H&E staining of colonic tissue. FFTZS effects on colonic length (B), villus length (C), crypt depth (D), and villus length‐to‐crypt depth ratio (E). FFTZS effects on MPO (F) and EPO (G) levels in colonic tissue, as well as serum IL‐10 (H) and IL‐17 (I) levels. Data are presented as mean ± SD (*n* = 6). ^#^ *p* < 0.05, ^##^ *p* < 0.01 versus CON group; *p < 0.05, **p < 0.01 versus MOD group.

Chemotherapy with 5‐FU and irinotecan is known to impair intestinal mucosal barrier function. To evaluate the protective effects of FFTZS against mucosal damage, H&E staining was performed to observe pathological changes in colonic tissues (Figure 2). The CON and FF‐C groups exhibited smooth and intact colonic mucosa, with tightly arranged epithelial cells and preserved villi structure. In contrast, the MOD group showed significant pathological damage, including villus shortening, crypt destruction, reduced numbers of goblet cells and glands, and inflammatory cell infiltration. Notably, the EI group displayed significant improvements, with reduced colonic damage compared with the MOD group. Villus length was significantly decreased in the MOD group compared with the CON group (*p* < 0.01). However, FFTZS pretreatment in the EI group significantly improved villus shortening (*p* < 0.05). No significant changes were observed in crypt depth among the treatment groups. The villus‐to‐crypt ratio (V/C), an indicator of absorptive capacity, was significantly lower in the MOD group compared with the CON group but was significantly increased in the EI group compared with the MOD group (Figure 2E). FFTZS also improved colonic length, which was significantly reduced in the MOD group compared with the CON group (*p* < 0.05; Figure 2B). These findings indicate that FFTZS intervention effectively mitigates chemotherapy‐induced colonic tissue damage.

### 3.3 FFTZS Reduces Inflammatory Infiltration in Colonic Tissues of Chemotherapy‐Treated Mice

We next evaluated the impact of FFTZS supplementation on inflammatory infiltration in colonic tissues. The activities of MPO and EPO were measured to assess neutrophil and eosinophil infiltration, respectively. There was no significant difference between the CON group and the FF‐C group. Compared with the CON group, the MOD group showed significantly increased MPO and EPO activities (*p* < 0.01; Figure 2F,G). However, these activities were significantly reduced in the EI and LI groups compared with the MOD group. Additionally, serum levels of DAO and D‐LA, markers of intestinal mucosal damage, were significantly elevated in the MOD group compared with the CON group. FFTZS intervention markedly alleviated these increases (Figure 2H,I).

### 3.4 FFTZS Prevents Mucus Layer Depletion in Colonic Tissues of Chemotherapy‐Treated Mice

**FIGURE 3:**
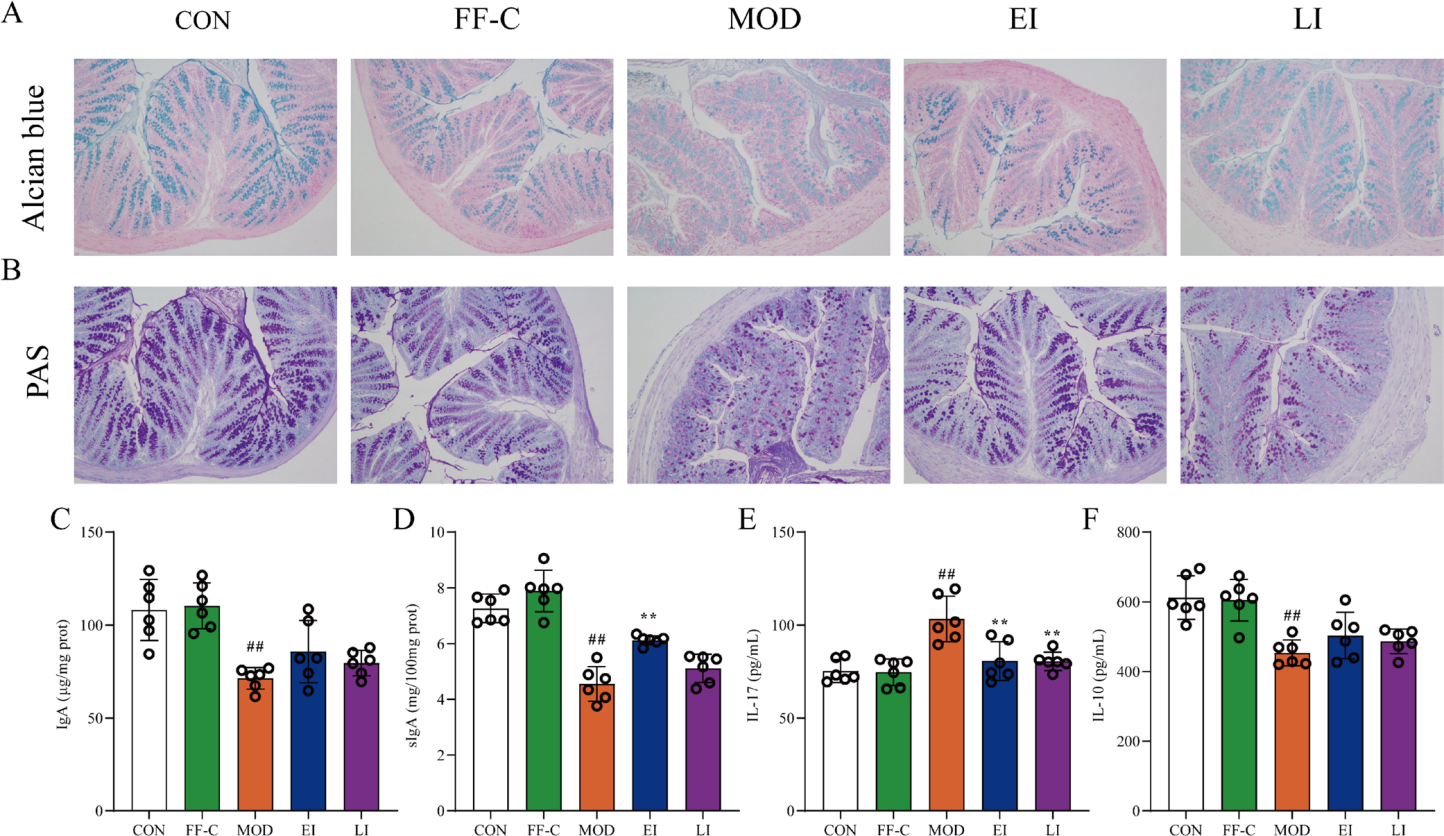
Effects of FFTZS on the colonic immune barrier in chemotherapy‐treated mice. FFTZS effects on (A) goblet cells and (B) mucus layer in colonic tissue. FFTZS effects on IgA (C) and sIgA (D) levels in colonic tissue, and serum IL‐10 (E) and IL‐17 (F) levels. Data are presented as mean ± SD (*n* = 6). ^##^ *p* < 0.01 versus CON group; **p < 0.01 versus MOD group.

Chemotherapy‐induced colitis is primarily caused by damage to intestinal epithelial cells, particularly goblet cells, which produce a protective mucus layer. To evaluate the effects of FFTZS on mucus layer integrity, Alcian blue staining was used to observe mucus in colonic tissues (Figure 3A). Compared with the CON group, the MOD group exhibited significant mucus layer damage following chemotherapy. However, the EI group showed notable improvements in mucus layer integrity compared with the MOD group. PAS staining further revealed a reduction in mucin‐producing goblet cells in colonic tissues of MOD mice, which was significantly restored following FFTZS treatment (Figure 3B).

We also evaluated intestinal immune function by measuring IgA and sIgA levels in colonic tissues (Figure 3C,D). sIgA levels were significantly increased in the FF‐C group compared with the CON group but were markedly reduced in the MOD group. Both the EI and LI groups showed significant increases in sIgA levels compared with the MOD group. Furthermore, inflammatory cytokine analysis revealed prominent inflammation in colonic tissues following chemotherapy with 5‐FU and irinotecan, which was significantly alleviated by FFTZS intervention. These findings suggest that FFTZS treatment enhances colonic immune function and protects against chemotherapy‐induced mucus layer depletion and goblet cell loss.

### 3.5 FFTZS Attenuates Apoptosis in Colonic Tissue Induced by Chemotherapy

**FIGURE 4:**
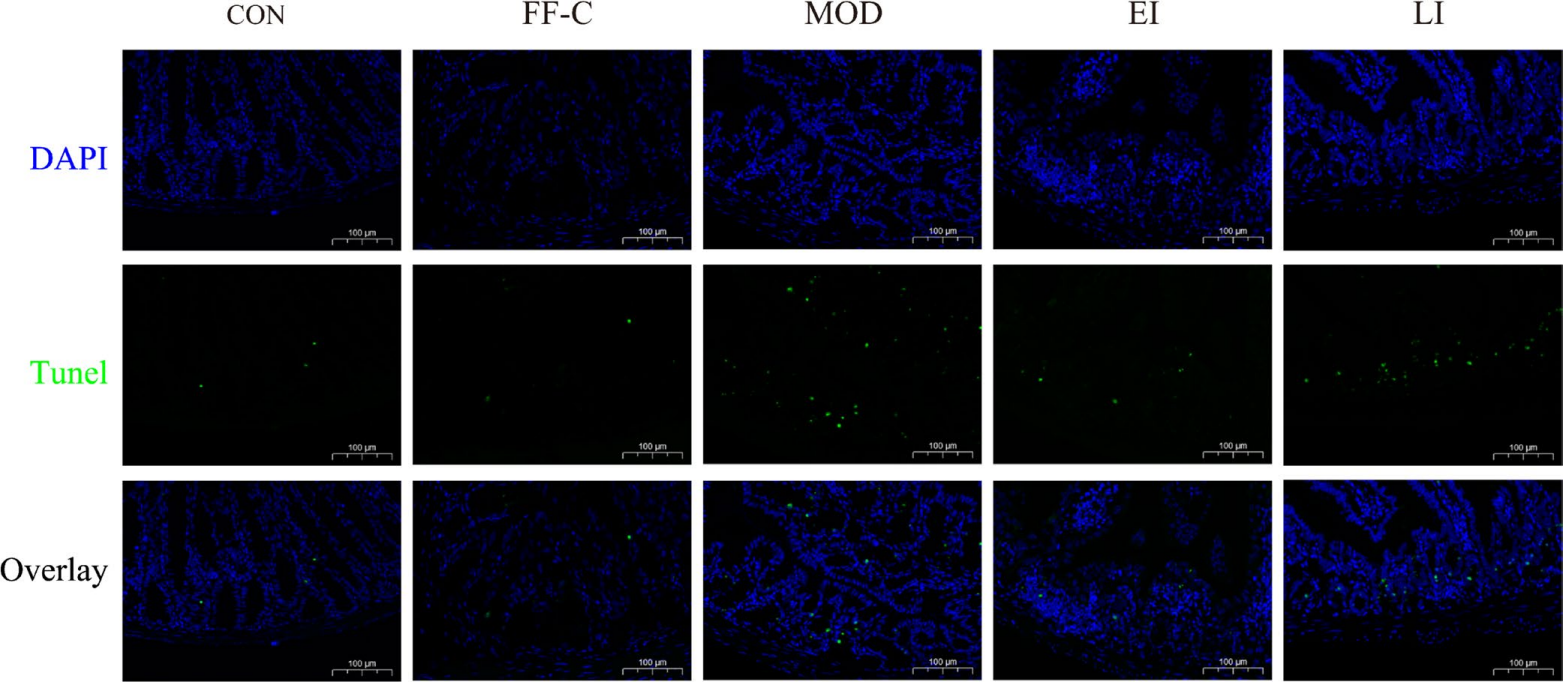
Effects of FFTZS on apoptosis in colonic tissue of chemotherapy‐treated mice.

To evaluate the effect of FFTZS on chemotherapy‐induced apoptosis in colonic tissue, TUNEL staining was performed on the colonic samples from each group (Figure 4). Quantitative analysis revealed that the MOD group exhibited a significant increase in the number of TUNEL‐positive cells compared to the CON group (*p* < 0.001). In contrast, prophylactic administration of FFTZS markedly reduced apoptosis in the EI group (^##^ *p* < 0.01 vs. MOD), while the LI group also showed a moderate but significant reduction (^#^ *p* < 0.05 vs. MOD). No significant differences were observed between the CON and FF‐C groups. These results suggest that FFTZS mitigates chemotherapy‐induced epithelial apoptosis, with more pronounced effects observed in early intervention.

### 3.6 FFTZS Promotes Expression of Tight Junction Proteins Claudin‐1 and ZO‐1 in Colonic Tissue

**FIGURE 5:**
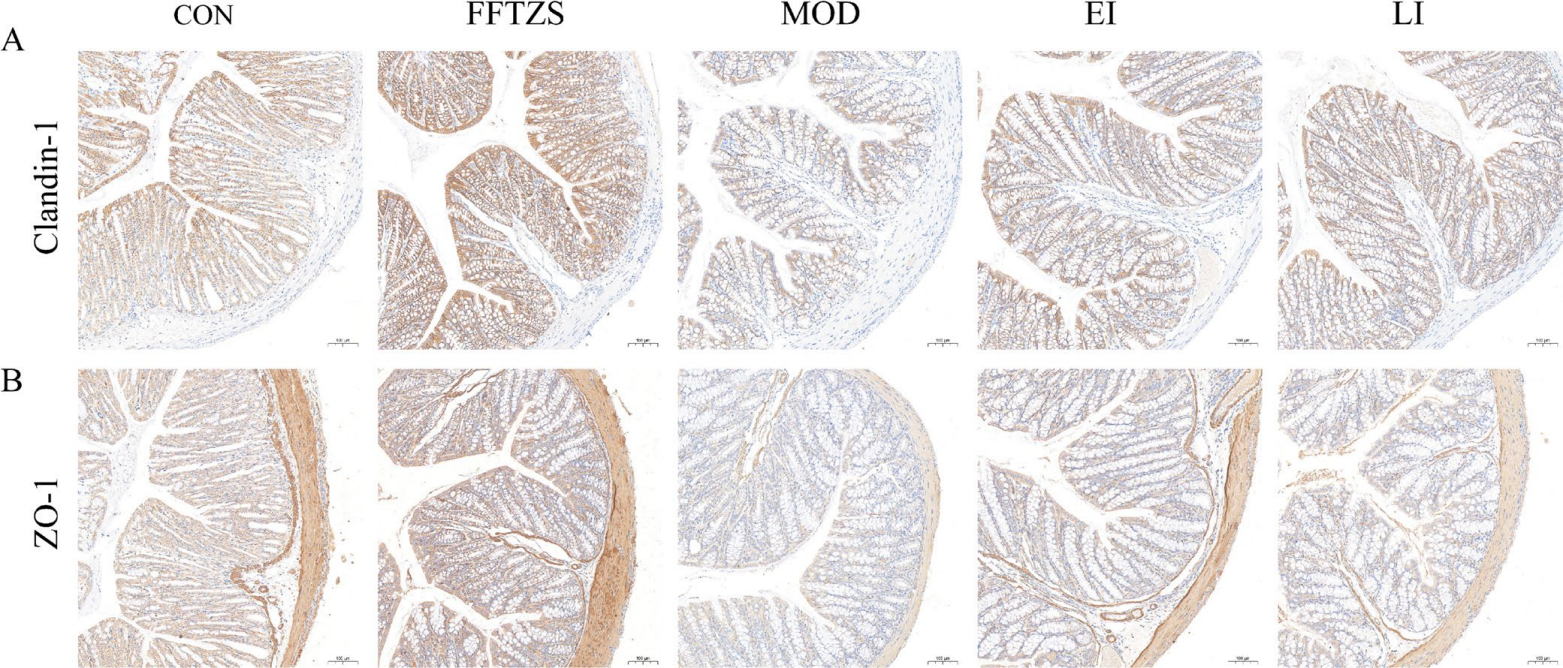
Effects of FFTZS on the expression of Claudin‐1 and ZO‐1 proteins in colonic tissue of chemotherapy‐treated mice.

Tight junction proteins and mucins play crucial roles in maintaining the stability of the intestinal mucosal barrier. Immunohistochemical staining was used to assess the expression levels of Claudin‐1 and ZO‐1 proteins in colonic tissues across all groups (Figure 5). The MOD group showed significantly reduced expression of Claudin‐1 and ZO‐1 compared to the CON group (*p* < 0.001), indicating disruption of the intestinal barrier by irinotecan and 5‐FU. In contrast, FFTZS treatment significantly restored the expression of both proteins. Specifically, the EI group demonstrated a robust increase in Claudin‐1 and ZO‐1 levels (^##^ *p* < 0.01 vs. MOD), while the LI group also exhibited significant improvements (^#^ *p* < 0.05 vs. MOD). These findings highlight the role of FFTZS in enhancing tight junction integrity and maintaining mucosal barrier function during chemotherapy.

### 3.7 FFTZS Modulates the Composition of Gut Microbiota in Chemotherapy‐Treated Mice

**FIGURE 6:**
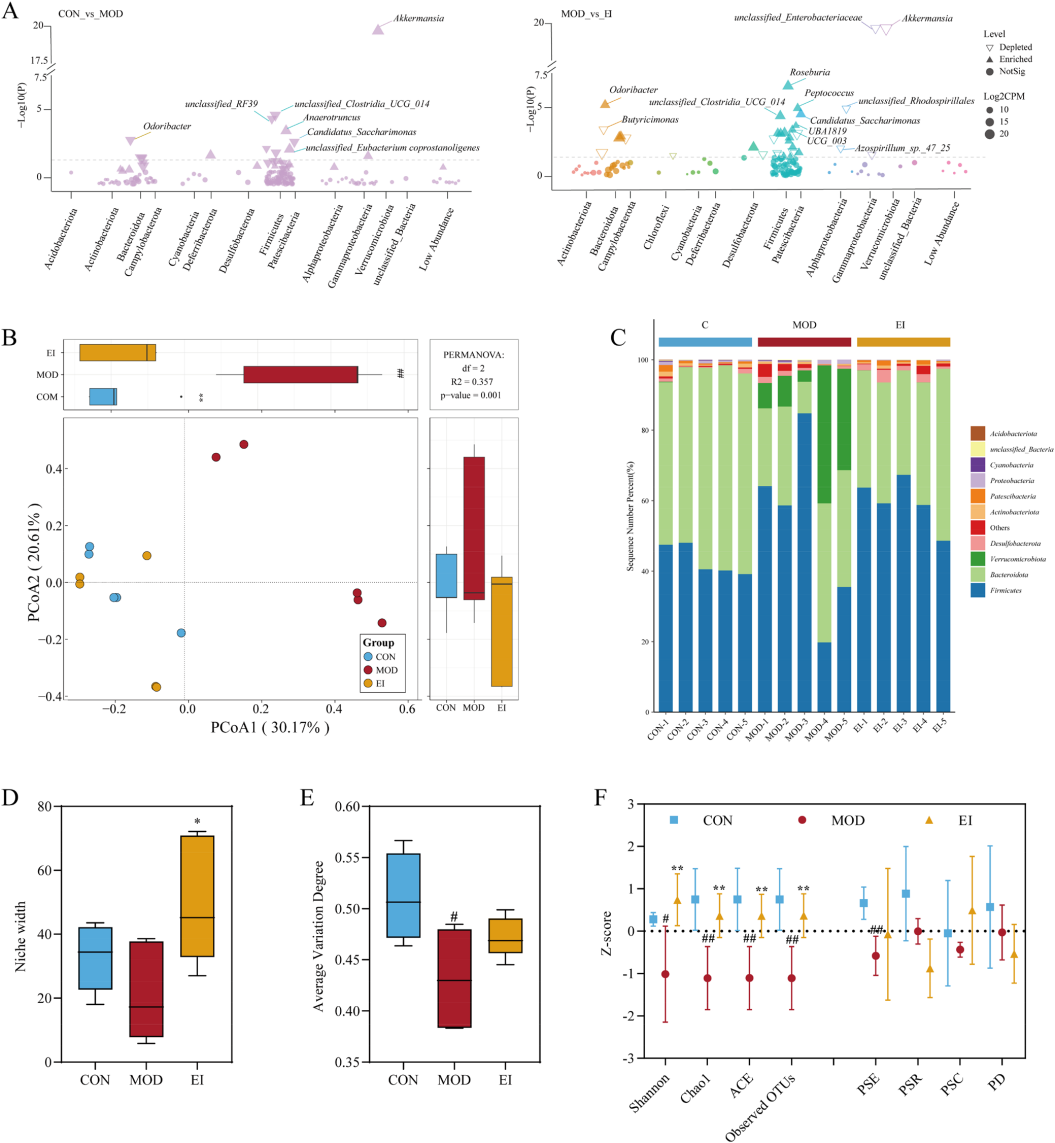
FFTZS improves gut microbiota composition in 5‐FU and irinotecan‐treated mice. (A) Manhattan plot showing enriched and depleted microbial taxa between different treatment groups. (B) Principal component analysis (PCA), with PCoA1 and PCoA2 representing the two most significant factors influencing microbial distribution. (C) Microbial community stacked bar plot. Analysis of microbial niche width (D) and average variation degree (E) among treatment groups. (F) Microbial diversity analysis. Data are presented as mean ± SD (*n* = 5). ^#^ *p* < 0.05, ^##^ *p* < 0.01 versus CON group; *p < 0.05, **p < 0.01 versus MOD group.

The effect of FFTZS on gut microbiota composition was analyzed using 16S rRNA sequencing. The structure of the gut microbiota in different groups is presented in Figure 6. Principal coordinates analysis (PCoA) revealed significant differences in the PCoA1 axis among the treatment groups. Compared to the MOD group, the PCoA1 value of the EI group was significantly increased (*p* < 0.01), indicating that FFTZS improved the β‐diversity of gut microbiota disrupted by irinotecan and 5‐FU. The first two principal coordinates, PCoA1 and PCoA2, accounted for 30.17% and 20.61% of the total variance, respectively, explaining a combined 50.78% of the variation in microbial communities. Notably, samples from the FFTZS groups clustered closer to those of the CON group than to the MOD group, suggesting that FFTZS treatment helps preserve or restore microbial homeostasis disrupted by chemotherapy. Alpha diversity indices, including Shannon, Chao1, ACE, and Pielou's evenness (PSE), were significantly reduced in the MOD group compared to the CON group (Figure 6F). Interestingly, FFTZS intervention partially restored these indices. FFTZS also increased the niche width of the gut microbiota, although it did not significantly alter the average variation degree.

Differential microbiota analysis using DESeq2 revealed that compared to the CON group, the MOD group showed significantly increased relative abundance of *Anaerotruncus*, *Akkermansia*, and *unclassified_* *Eubacterium coprostanoligenes* , while *unclassified_Clostridia_UCG_014*, *unclassified_RF39*, *Candidatus_Saccharimonas*, and *Odoribacter* were significantly decreased. FFTZS treatment in the EI group notably increased the relative abundance of *Roseburia*, *Peptococcus*, *Odoribacter*, *unclassified_Clostridia_UCG_014*, *Candidatus_Saccharimonas*, and *UBA1819*, while reducing the abundance of *Akkermansia*, *unclassified_Enterobacteriaceae*, *unclassified_Rhodospirillales*, *Butyricimonas*, and *Azospirillum_sp._47_25*. These results indicate that FFTZS reshapes the gut microbiota composition disrupted by chemotherapy.

To validate the changes observed in 16S rRNA sequencing, we performed qPCR to assess the relative abundance of *Akkermansia* and *Roseburia* in mouse fecal samples. As shown in Figure S1, the abundance of *Akkermansia* was significantly higher in the MOD group than in the CON group (*p* < 0.01), while FFTZS treatment markedly reduced its level (*p* < 0.01) (Figure S1A). In contrast, the abundance of *Roseburia* showed no significant change in the MOD group but was significantly increased following FFTZS intervention (*p* < 0.01) (Figure S1B). These results are consistent with the 16S rRNA sequencing data and further confirm the modulatory effects of FFTZS on specific beneficial gut bacteria.

### 3.8 FFTZS Alleviates Metabolic Dysregulation in the Gut Microbiota of Chemotherapy‐Treated Mice

Fecal metabolomic analysis was conducted using UPLC‐HRMS to evaluate the impact of FFTZS intervention. Orthogonal partial least squares discriminant analysis (OPLS‐DA) revealed distinct metabolic profiles among the groups. The group difference scores were 47.4% between the MOD and CON groups, 40.9% between the EI and MOD groups, and only 27.3% between the EI and CON groups. These results suggest that FFTZS intervention shifted the metabolic profile of chemotherapy‐treated mice closer to that of the CON group.

**FIGURE 7:**
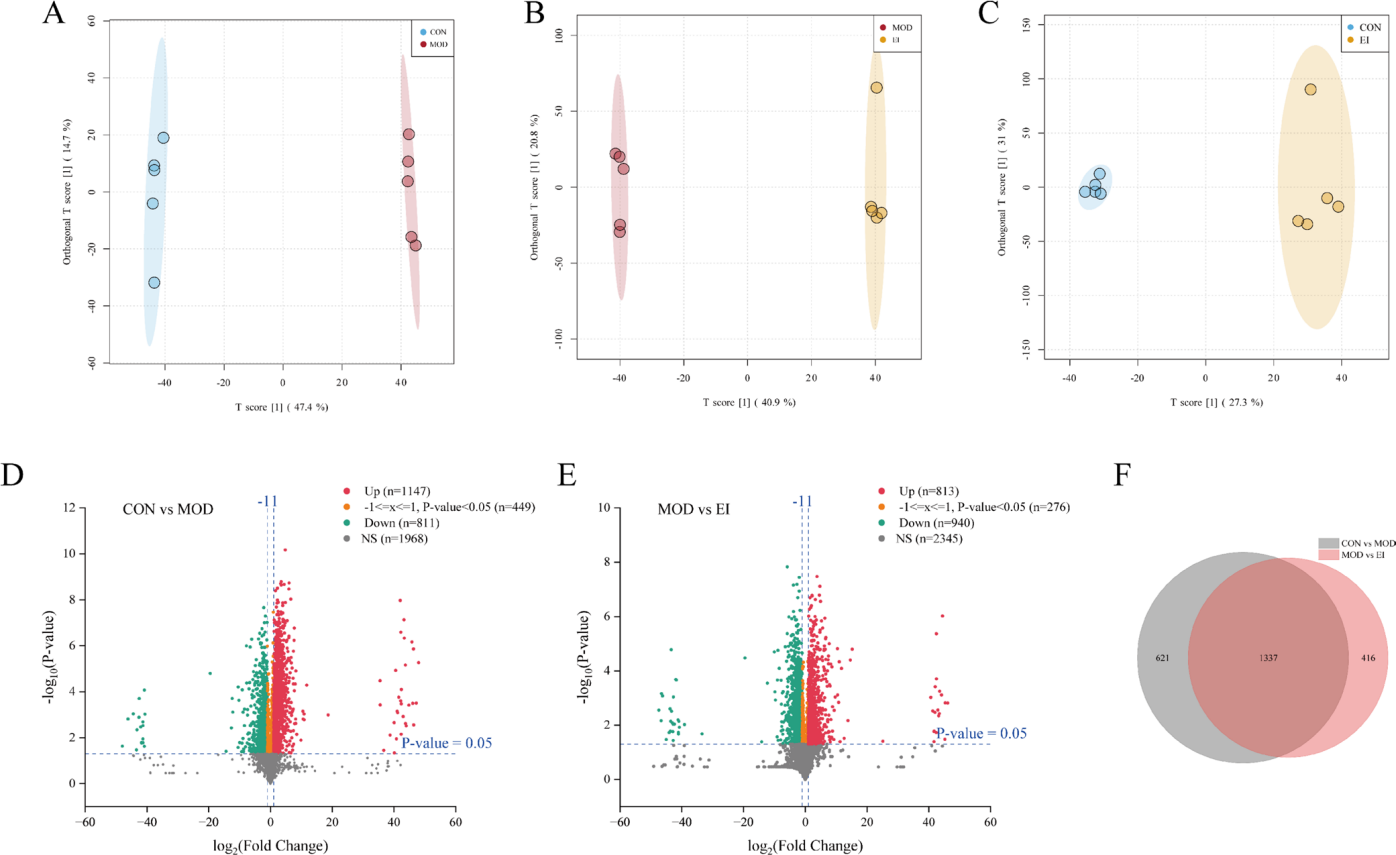
Effects of FFTZS on the metabolic profile of gut microbiota in chemotherapy‐treated mice. (A) OPLS‐DA score plot for CON versus MOD, (B) MOD versus EI, and (C) CON versus EI. (D) Volcano plot of differential metabolites between CON and MOD groups. (E) Volcano plot of differential metabolites between MOD and EI groups. (F) Venn diagram of overlapping differential metabolites.

Volcano plots based on log_2_ fold changes showed that irinotecan and 5‐FU treatment led to the downregulation of 811 metabolites and upregulation of 1147 metabolites compared to the CON group. FFTZS intervention resulted in the downregulation of 940 metabolites and upregulation of 817 metabolites compared to the MOD group, with 1337 shared differential metabolites (Figure 7F).

**FIGURE 8:**
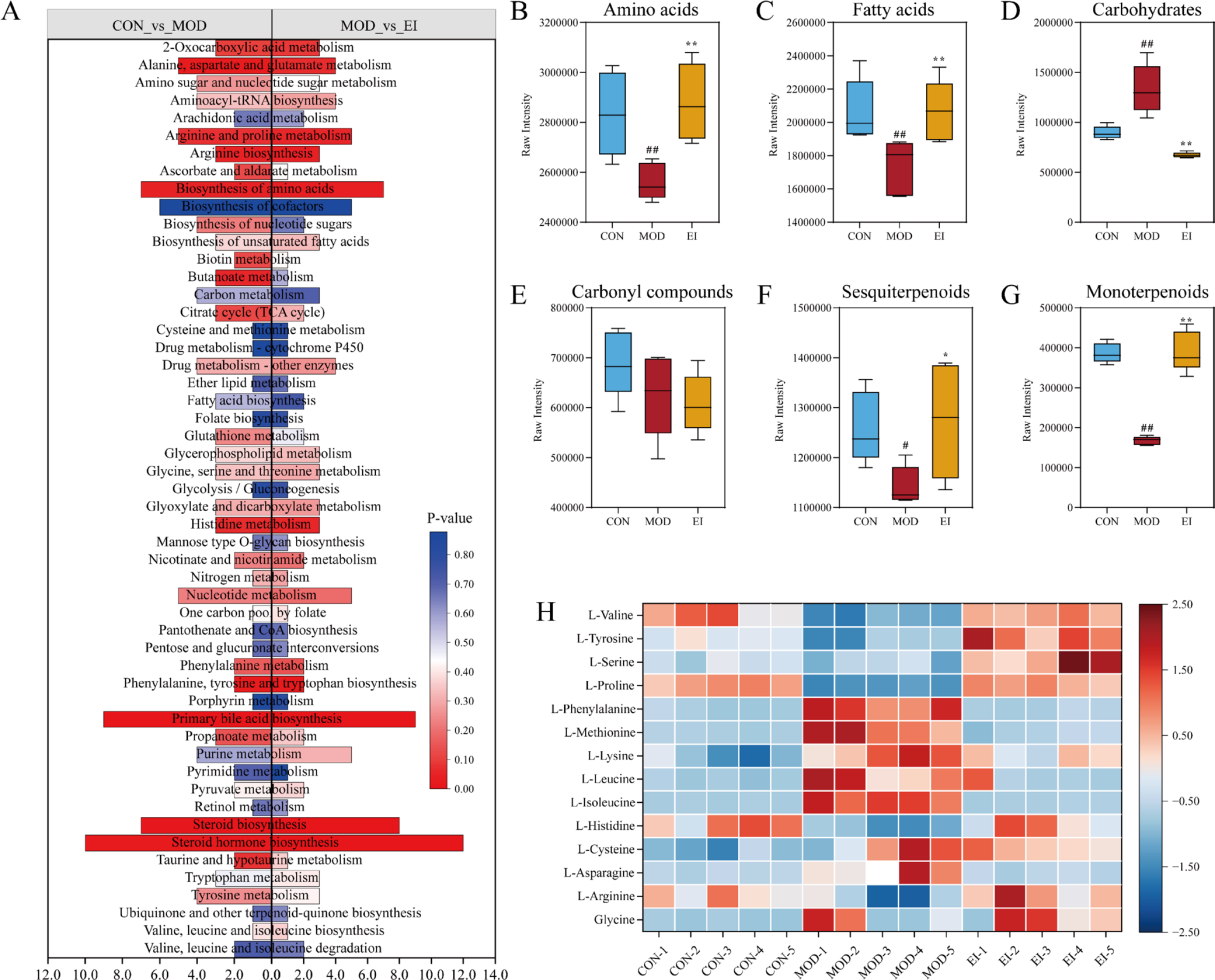
FFTZS alleviates metabolic disturbances in the gut microbiota of chemotherapy‐treated mice. (A) KEGG enrichment pathway analysis based on metabolomics. FFTZS effects on fecal concentrations of (B) amino acids, (C) fatty acids, (D) carbohydrates, (E) carbonyl compounds, (F) sesquiterpenoids, and (G) monoterpenoids. (H) Heatmap of 14 amino acid metabolites among different treatment groups. #*p* < 0.05, ##*p* < 0.01 versus CON group; *p < 0.05, **p < 0.01 versus MOD group.

KEGG pathway enrichment analysis revealed that FFTZS treatment significantly impacted metabolic pathways related to amino acid biosynthesis and metabolism, including alanine, aspartate, and glutamate metabolism; biosynthesis of amino acids; arginine and proline metabolism; histidine metabolism; and steroid biosynthesis. Further analysis of metabolite classes, including amino acids, fatty acids, carbohydrates, carbonyl compounds, sesquiterpenoids, and monoterpenoids, showed various degrees of improvement following FFTZS intervention, except for carbonyl compounds (Figure 8A–G).

Heatmap analysis of the relative abundance of 14 amino acids revealed that FFTZS intervention in the EI group restored the levels of several amino acids disrupted in the MOD group, making them closer to those in the CON group (Figure 8H). These findings suggest that FFTZS may alleviate chemotherapy‐induced intestinal mucosal injury by modulating amino acid metabolism pathways.

### 3.9 Results of KEGG Pathway Enrichment Analysis

The KEGG pathway enrichment analysis (Figure 8A) revealed significant enrichment of multiple amino acid metabolism‐related pathways in the MOD group compared to the CON group. These pathways included biosynthesis of amino acids, histidine metabolism, and alanine, aspartate, and glutamate metabolism, suggesting abnormal amino acid metabolism in the MOD group. Additionally, pathways associated with bile acid and steroid metabolism, such as primary bile acid biosynthesis, steroid biosynthesis, and steroid hormone biosynthesis, were significantly enriched, indicating potential disruptions in bile acid and steroid hormone metabolism. Notably, comparisons between the MOD and EI groups showed varying degrees of improvement in these aberrant metabolic pathways, suggesting that EI intervention may regulate amino acid, bile acid, and steroid metabolism.

Further analysis of the levels of amino acids, fatty acids, carbohydrates, carbonyl compounds, sesquiterpenoids, and monoterpenoids across treatment groups yielded results shown in Figure 8B–G. Compared to the CON group, the MOD group exhibited significantly reduced levels of amino acids, fatty acids, sesquiterpenoids, and monoterpenoids (*p* < 0.01), while carbohydrate levels were significantly elevated (*p* < 0.01). These changes suggest that 5‐FU and irinotecan‐induced damage to the intestinal mucosa may be associated with disruptions in metabolic equilibrium. Importantly, the EI group demonstrated significant improvements in these abnormal metabolite levels. Compared to the MOD group, the EI group showed significantly increased levels of amino acids, fatty acids, sesquiterpenoids, and monoterpenoids (*p* < 0.01) and reduced carbohydrate levels (*p* < 0.01), approaching those observed in the CON group. This indicates that EI intervention effectively reverses the metabolic dysregulation induced by 5‐FU and irinotecan, offering potential protective effects.

Figure 8H illustrates the relative abundance of various amino acids across treatment groups. The CON group exhibited baseline levels with minimal variation in amino acid expression. In contrast, the MOD group displayed significant upregulation or downregulation of several amino acids, highlighting pronounced metabolic disturbances caused by 5‐FU and irinotecan. Specifically, levels of L‐methionine, L‐phenylalanine, and L‐isoleucine were significantly elevated, while levels of L‐proline, L‐tyrosine, and L‐valine were significantly reduced. Remarkably, amino acid levels in the EI group tended to revert to those of the CON group, suggesting that EI intervention ameliorates the amino acid metabolic disturbances caused by chemotherapy.

### 3.10 Correlation Between Differential Microbiota and Amino Acids

**FIGURE 9:**
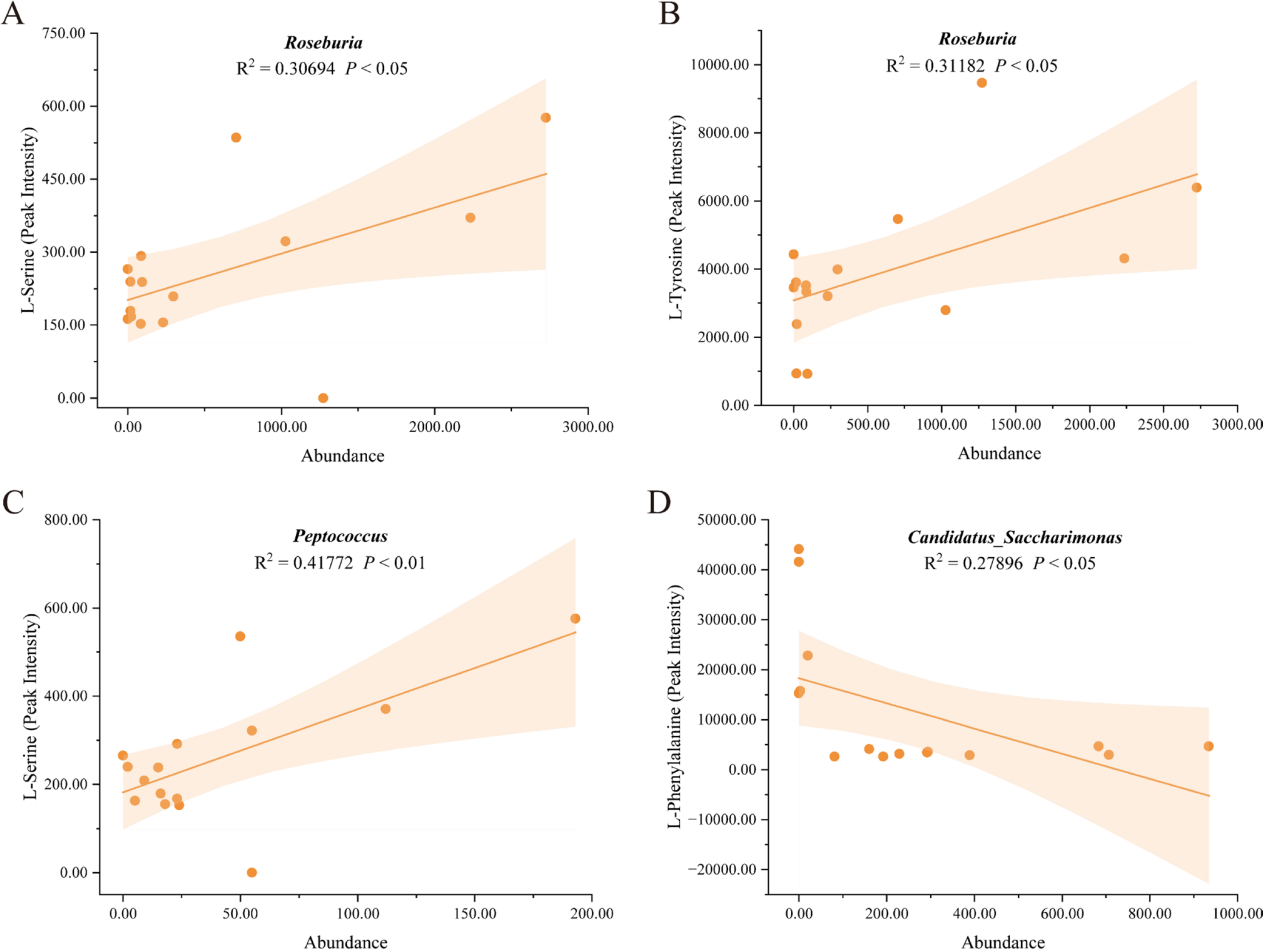
Correlation analysis between gut microbiota and amino acid metabolism. Correlations of *Roseburia* with L‐serine (A) and L‐tyrosine (B), *Peptococcus* with L‐serine (C), and *Candidatus Saccharimonas* with L‐phenylalanine (D).

Using Pearson correlation analysis, we explored potential associations between differential microbiota and amino acids. The results (Figure 9) showed significant positive correlations between *Roseburia* and both L‐serine (*R* ^2^ = 0.30694, *p* < 0.05) and L‐tyrosine (*R* ^2^ = 0.31182, *p* < 0.05), indicating that *Roseburia* may play a role in regulating these amino acids. Similarly, *Peptococcus* displayed a significant positive correlation with L‐serine (*R* ^2^ = 0.41772, *p* < 0.01). Conversely, *Candidatus_Saccharimonas* exhibited a significant negative correlation with L‐phenylalanine (*R* ^2^ = 0.27896, *p* < 0.05), suggesting its potential involvement in aromatic amino acid metabolism. These significant correlations provide important insights into the biological roles of microbiota in amino acid metabolism.

### 3.11 Integration of Gut Microbiota Composition With Biochemical and Physiological Indicators

**FIGURE 10:**
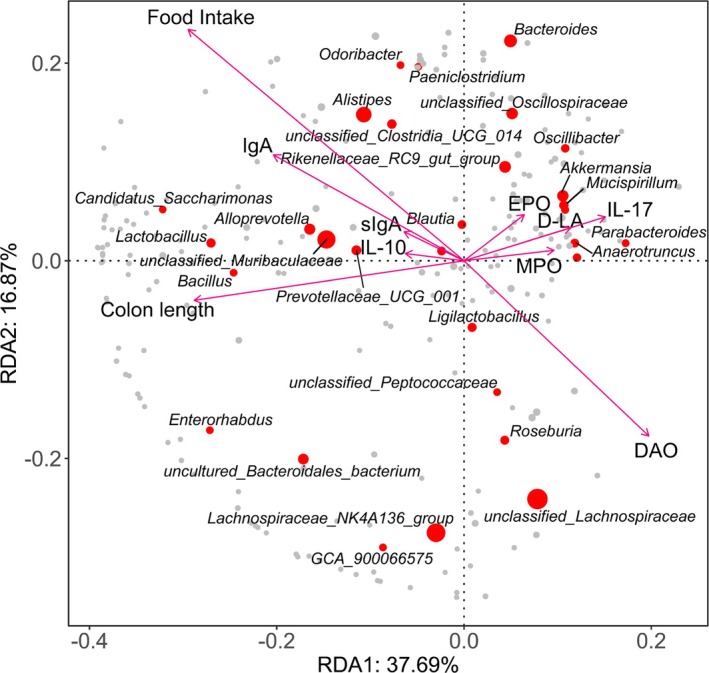
Redundancy analysis of gut microbiota composition and host metabolic parameters.

We further investigated the relationship between gut microbiota composition and biochemical markers (DAO, D‐LA, EPO, MPO, IgA, sIgA, IL‐10, IL‐17) as well as physiological parameters (food intake and colon length) using redundancy analysis (RDA; Figure 10). The RDA plot quantified the influence of these indicators on bacterial community composition. DAO levels, food intake, and colon length emerged as primary drivers of microbiota variations. Notably, *Alistipes*, *Odoribacter*, *Alloprevotella*, *unclassified_Muribaculaceae*, and *Candidatus_Saccharimonas* were positively correlated with food intake and IgA but negatively correlated with DAO levels. Conversely, *Akkermansia* and *Mucispirillum* were positively associated with EPO, D‐LA, and IL‐17. These findings underscore the complex interplay between gut microbiota, metabolic health, and immune regulation in the context of chemotherapy‐induced intestinal damage.

## 4 Discussion

In this study, we demonstrated that prophylactic administration of FFTZS alleviated chemotherapy‐induced anorexia and diarrhea caused by irinotecan and 5‐FU. However, FFTZS failed to prevent weight loss in the treated mice. FFTZS improved histopathological damage, increased the thickness of the mucus layer, and elevated sIgA levels in intestinal tissues, thereby enhancing the immune function of the colonic tissue. Furthermore, it promoted the expression of tight junction proteins in the colon, which helped maintain intestinal barrier integrity and suppressed apoptosis in colonic tissues. FFTZS also increased the relative abundance of beneficial bacteria and reduced the relative abundance of opportunistic pathogens in the colon, leading to alterations in microbial metabolites, particularly those associated with amino acid metabolism. Collectively, these findings suggest that FFTZS mitigates chemotherapy‐induced intestinal mucosal damage by improving intestinal barrier function, modulating gut microbiota, and regulating amino acid metabolism. These results provide a promising basis for the prospective application of FFTZS in mitigating adverse clinical reactions to chemotherapy.

The combination of TCM formulations with chemotherapy has been a focus of research in cancer treatment. Although chemotherapy is a common strategy for cancer management, it is often associated with substantial adverse side effects (Zhou et al. 2024). Studies have shown that administering TCM formulations prior to chemotherapy can help alleviate these side effects. For instance, pretreatment with 2′‐fucosyllactose has been shown to prevent weight loss caused by 5‐FU and to improve inflammation scores, reduce pro‐inflammatory cytokine production, and mitigate epithelial apoptosis and disruption of tight junction complexes. Interestingly, its synchronous administration during chemotherapy was less effective in ameliorating intestinal mucositis than pretreatment (Zhao et al. 2022). Similarly, sulforaphane administered 5 days before 5‐FU treatment was found to alleviate histopathological damage, reduce weight loss, mitigate intestinal inflammation, and improve intestinal permeability (Wei et al. 2020). Preventive administration of naringin significantly inhibited oxidative stress, inflammation, and apoptosis in the hearts of rats exposed to 5‐FU (Gui et al. 2023). In 5‐FU‐induced mucositis, fructo‐oligosaccharides pretreatment maintained the expression of tight junction proteins, reduced inflammatory infiltration and histological scores, and improved SCFA production (Carvalho et al. 2021). Notably, our findings suggest that the FFTZS‐treated EI group outperformed the LI group in food intake, diarrhea index, villus length, and sIgA levels, indicating that the benefits of FFTZS are more pronounced when treatment begins at least 1 week before chemotherapy. These results emphasize the importance of incorporating TCM interventions early in treatment plans, as patients may gain greater benefits and experience fewer side effects during chemotherapy.

Several studies have reported the effects and mechanisms of FFTZS components in cancer chemotherapy. For instance, polysaccharides derived from *Pseudostellaria heterophylla* can enhance cellular immunity by improving macrophage phagocytosis and promoting spleen cell proliferation. These polysaccharides also regulate the gut microbiota by increasing beneficial microbes like *Odoribacter* and *Mucispirillum*, thereby mitigating chemotherapy‐induced immunosuppression. Ganoderic acids from *Ganoderma lucidum* alleviate 5‐FU‐induced peripheral fatigue‐like behavior by improving muscle mass and mitochondrial function, increasing glycogen levels and ATP production, reducing lactate levels and LDH activity, and suppressing the expression of p‐AMPK, IL‐6, and TNF‐α (Abulizi et al. 2021). Furthermore, fungal immunomodulatory proteins from *Ganoderma lucidum* can restore claudin‐1 expression to reduce irinotecan‐induced intestinal damage (Li, Chu, et al. 2023). Polysaccharides from *Poria cocos* exhibit prebiotic activity, alleviating 5‐FU‐induced adverse effects in mice and improving treatment efficacy (Yin et al. 2022). They also ameliorate weight loss and enhance intestinal barrier function by strengthening tight junction proteins and adhesion molecules while increasing the relative abundance of beneficial fecal bacteria such as *Bacteroides acidifaciens* and *Bacteroides intestinihominis*. Polyphenolic compounds from *Crataegus pinnatifida* have been shown to promote autophagy and cell cycle arrest in HepG2 and Hep3B cells, leading to increased apoptosis (Guo et al. 2018, 2019). Meanwhile, *Hordei Fructus Germinatus* and *Oryzae Fructus Germinatus* are traditional remedies for functional dyspepsia and are commonly included in TCM prescriptions for post‐chemotherapy management (Liu, Chen, et al. 2023; Zhang et al. 2021).

The intestinal barrier comprises physical, chemical, immune, and microbial barriers. Key parameters reflecting the physical barrier include intestinal morphology, tight junctions, and permeability. Intestinal morphology indicators, such as villus length, crypt depth, and their ratio, provide insights into the integrity of the intestinal barrier. In our study, prophylactic administration of FFTZS significantly increased villus length and the villus‐to‐crypt ratio in the colon, suggesting improved intestinal morphology in chemotherapy‐treated mice. Chemotherapy‐induced apoptosis of intestinal epithelial cells is a primary driver of villus shortening. In the irinotecan and 5‐FU chemotherapy groups, a marked increase in apoptosis was observed, which was alleviated by FFTZS prophylactic treatment. However, apoptosis remained prominent in the LI group.

In addition to epithelial cells, tight junctions between cells play a crucial role in forming and maintaining the physical barrier of the intestinal epithelium (Liebing et al. 2024). These junctions prevent harmful substances, such as bacteria and endotoxins, from translocating across the mucosa into the bloodstream. Tight junctions consist of transmembrane proteins (e.g., claudin‐1) and scaffolding proteins (e.g., ZO‐1), which help maintain intestinal permeability. Extensive research has shown that irinotecan and 5‐FU disrupt tight junctions and stimulate pro‐inflammatory cytokine secretion, leading to impaired intestinal barrier function. Downregulation or reduced activity of ZO‐1 hinders tight junction formation, compromising mucosal barrier function and facilitating bacterial and toxin translocation, thus increasing the risk of intestinal infections (Oshima and Miwa 2016). Claudin‐1 regulates epithelial polarity, intestinal permeability, and mucosal integrity, playing an essential role in maintaining epithelial function and preventing toxin entry (Bai, Liu, et al. 2024). Our study revealed that FFTZS supplementation significantly enhanced the expression of Claudin‐1 and ZO‐1, corresponding to changes in serum D‐LA levels. These findings further confirm the enhanced intestinal physical barrier function under FFTZS treatment.

D‐LA and DAO are specific biomarkers reflecting intestinal barrier integrity (Cheng et al. 2024; Li, Huang, et al. 2023; Ouyang et al. 2023). D‐LA, a metabolic product of intestinal bacteria, enters the bloodstream through damaged mucosa when intestinal permeability increases, making serum D‐LA levels a critical indicator of barrier dysfunction. DAO, an intracellular enzyme in intestinal epithelial cells, is released upon mucosal damage, and its serum concentration reflects epithelial injury. Consistent with previous reports, chemotherapy‐treated groups exhibited significant elevations in serum D‐LA and DAO levels. FFTZS treatment significantly reduced D‐LA levels; although, DAO levels showed no notable changes. These results indicate that while FFTZS enhances the physical barrier function of the intestine, epithelial cell damage remains a persistent concern.

The mucus layer of the gut chemical barrier plays a critical role in maintaining intestinal health. This layer, composed of mucins secreted by goblet cells, acidic mucopolysaccharides, glycoproteins, and antimicrobial peptides, serves as the first line of defense against harmful agents. In addition to physically covering and protecting intestinal epithelial cells, the mucus layer provides chemical defense against pathogens and regulates immune responses through its diverse components. Studies have demonstrated that deficiencies in the mucus layer are directly associated with impaired gut barrier function and systemic inflammation (Ye et al. 2021). Consistent with the literature, our observations in irinotecan‐ and 5‐FU‐treated mice reveal damage to the gut chemical barrier, primarily manifesting as reduced goblet cell numbers and a thinner mucus layer. Notably, supplementation with FFTZS, especially as a prophylactic intervention, significantly mitigated goblet cell depletion and maintained the integrity of the mucus layer during chemotherapy.

The intestinal immune barrier, comprising intestinal lymphocytes, immune cells, associated cytokines, and sIgA, is a key component of the intestinal barrier and plays an essential role in maintaining normal gut barrier function. In this study, we observed that intervention with FFTZS significantly downregulated the pro‐inflammatory cytokine IL‐17 in colonic mucosa while markedly increasing sIgA levels, indicating an improvement in gut immune barrier function. In line with our findings, previous research has shown that *Pseudostellaria heterophylla* polysaccharides can restore chemotherapy‐induced intestinal mucositis by improving gut morphology, modulating immune cell homeostasis, and alleviating intestinal inflammation. Additionally, accumulating evidence indicates that *Ganoderma lucidum* polysaccharides possess anti‐inflammatory and immunomodulatory properties. Previous studies have demonstrated that these polysaccharides significantly suppress the secretion of TNF‐α, IL‐1β, IL‐6, IL‐17A, and IL‐4 in lamina propria lymphocytes, enhance B cell differentiation, and reduce the proportions of Th17, NK, and NKT cells (Wei et al. 2018).

The intestinal microbial barrier, constituted by a stable microbiota ecosystem, is crucial for the development and regulation of immune functions (Liu, Duan, et al. 2023; Meng et al. 2023). Our study found that FFTZS treatment significantly increased the relative abundance of *Roseburia*, *Peptococcus*, *Odoribacter*, *unclassified_Clostridia_UCG_014*, *Candidatus_Saccharimona*, and *UBA1819*, while reducing the relative abundance of *Akkermansia*, *unclassified_Enterobacteriaceae*, *unclassified_Rhodospirillales*, *Butyricimonas*, and *Azospirillum_sp._47_25*. Notably, *Akkermansia*, a mucin‐degrading microbial member, has garnered attention for its potential health benefits, particularly in promoting gut mucosal integrity and improving metabolic states. However, under conditions of chemotherapy‐induced malnutrition, the competitive advantage of *Akkermansia* may shift (Zhang et al. 2019). Chemotherapy often leads to a significant reduction in food intake, causing nutrient deficiencies in the gut. Under these conditions, *Akkermansia* gains energy by enhancing its mucin‐degradation capabilities, thereby achieving dominance within the microbiota. This alteration may exacerbate mucosal damage, weaken barrier function, and worsen intestinal inflammation and nutrient absorption disorders (Mo et al. 2024). Our findings suggest that FFTZS intervention substantially reduces *Akkermansia* abundance, potentially protecting the host by mitigating mucin degradation and facilitating mucosal repair. Additionally, FFTZS significantly increased the relative abundance of beneficial microbes such as *Roseburia*, *Peptococcus*, and *Odoribacter*. *Roseburia* and its metabolites have been shown to prevent intestinal inflammation, improve gut permeability, and enhance tight junction protein expression, thereby restoring gut barrier function (Kang et al. 2023). *Odoribacter* is positively associated with short‐chain fatty acid production, amino acid metabolism, and AMPK signaling. These bacteria produce SCFAs, such as butyrate and propionate, which enhance gut barrier function, modulate immune responses, and suppress inflammation.

In this study, we observed significant disruptions in metabolite levels in mice treated with irinotecan and 5‐FU, including aberrations in amino acids, fatty acids, carbohydrates, and bile acid metabolites. This aligns with existing literature indicating that chemotherapeutic agents, such as 5‐FU, not only kill tumor cells through cytotoxic mechanisms but also cause widespread disturbances in host metabolic homeostasis (Teng et al. 2024; Wang et al. 2024). Elevated carbohydrate levels may reflect the activation of glycolysis and gluconeogenesis pathways to meet energy demands, although such metabolic imbalances often coincide with aberrant lipid and protein metabolism, leading to decreased metabolic efficiency. FFTZS treatment significantly increased amino acid and fatty acid levels while restoring carbohydrate levels closer to normal, suggesting that FFTZS may reshape metabolic pathways by promoting lipid and protein metabolism while inhibiting excessive carbohydrate metabolism, thereby alleviating chemotherapy‐induced metabolic disruptions. Furthermore, the significant increase in monoterpenoid and sesquiterpenoid levels in the FFTZS‐treated group indicates a potential role in modulating oxidative stress. These compounds, previously reported to possess antioxidant, anti‐inflammatory, and immunomodulatory properties (Bartikova et al. 2014), may contribute to alleviating chemotherapy‐induced oxidative stress and inflammatory responses, ultimately improving intestinal metabolic homeostasis.

This study demonstrates that the expression levels of various amino acids were significantly altered in the MOD group, indicating marked amino acid metabolic disturbances induced by 5‐FU and irinotecan treatment. These disruptions are likely related to chemotherapy‐induced intestinal injury, enhanced protein catabolism, and increased energy demands, reflecting adaptive metabolic changes under stress conditions (Xin et al. 2024). Notably, FFTZS intervention markedly alleviated these metabolic abnormalities, restoring most amino acid levels to those observed in the normal control group, which may play a crucial role in the recovery of intestinal barrier function. Amino acids are essential for the growth of intestinal epithelial cells and the maintenance of mucosal integrity, contributing to the suppression of oxidative stress and reduction of pro‐inflammatory cytokine levels. Research has shown that serine enhances the expression of tight junction proteins in the jejunum and ileum, mitigates intestinal apoptosis, inflammation, and oxidative stress, and reduces diarrhea incidence (Zhou et al. 2018). Similarly, phenylalanine inhibits ROS‐induced oxidative damage and apoptosis, enhances tight junction protein expression, and downregulates pro‐inflammatory cytokine levels (Feng et al. 2015, 2017).

Our findings revealed significantly elevated serine and phenylalanine levels in FFTZS‐treated mice compared to the MOD group, alongside a notable increase in tight junction protein expression (ZO‐1 and Claudin‐1). This suggests that FFTZS may improve intestinal barrier integrity by modulating amino acid metabolic pathways and influencing tight junction protein expression. Further analysis indicated that FFTZS‐enriched bacterial genera, such as *Roseburia* and *Peptococcus*, were closely associated with specific amino acid levels. *Roseburia* showed positive correlations with L‐serine and L‐tyrosine, while *Peptococcus* correlated positively with L‐serine, implying that these genera may directly or indirectly contribute to the restoration of metabolic balance through amino acid metabolism (He et al. 2018). Additionally, the negative correlation observed between the enrichment of *Candidatus Saccharimonas* and L‐phenylalanine may suggest its potential role in regulating aromatic amino acid metabolism. These results highlight that FFTZS could enhance amino acid metabolic homeostasis through gut microbiota modulation.

While this study systematically reveals the protective effects of FFTZS against 5‐FU‐ and irinotecan‐induced intestinal mucositis in mice, several limitations warrant further investigation. First, this study focused on intestinal tissue protection, but chemotherapeutic agents may also induce systemic toxicities affecting other organs, such as the liver, heart, or bone marrow. The potential benefits of FFTZS in mitigating these systemic side effects require further exploration. Second, although this study examined the roles of FFTZS in modulating gut microbiota, amino acid metabolism, and intestinal barrier function, the precise molecular mechanisms, particularly those related to microbiota–metabolite–host interactions, remain to be fully elucidated. Moreover, this study evaluated only a single dose (2.5 g/kg) of FFTZS, which was selected based on preliminary screening that identified it as the optimal dose for ameliorating chemotherapy‐induced intestinal injury in mice. Nonetheless, the absence of a comprehensive dose–response analysis limits the pharmacological interpretation of FFTZS's efficacy and safety range. Future studies are warranted to systematically explore the therapeutic window and dose‐dependent effects of FFTZS in preclinical models, which will be critical for guiding its clinical application.

Additionally, while the murine model of 5‐FU and irinotecan‐induced intestinal mucositis is widely used and enables controlled investigation of intestinal barrier damage, microbiota dysbiosis, and metabolic alterations, it does not fully capture the complexity of human mucositis. Differences in gut microbiome composition, host immunity, chemotherapy regimens, nutritional status, and drug pharmacokinetics between mice and humans may affect both disease manifestation and treatment response. Moreover, the psychological and systemic effects of chemotherapy, which contribute to mucositis severity in cancer patients, are difficult to model in rodents. Therefore, while our findings provide a foundational understanding of FFTZS's protective mechanisms, further validation in human‐relevant systems—such as patient‐derived organoids, humanized microbiota models, or early‐phase clinical trials—is essential to confirm its translational potential.

The findings of this study offer important implications for clinical practice. Chemotherapy‐induced intestinal mucositis remains a significant challenge in oncology, often necessitating dose reductions or treatment interruptions that compromise therapeutic efficacy. FFTZS, as a TCM formula composed of *Pseudostellaria heterophylla*, *Ganoderma lucidum*, and other herbs, demonstrates multi‐targeted protective effects by modulating intestinal inflammation, barrier integrity, and microbiota metabolism. The observed benefits, particularly with prophylactic administration, suggest its potential role as an adjunctive therapy to prevent mucositis in patients undergoing irinotecan‐ and 5‐FU‐based regimens. Given its oral formulation, historical safety profile, and immunomodulatory properties, FFTZS may serve as a viable complementary approach in integrative cancer care. Nonetheless, clinical trials are warranted to confirm its efficacy and safety in human populations and to explore optimal dosing regimens and timing relative to chemotherapy administration.

## 5 Conclusion

In conclusion, our findings demonstrate that FFTZS confers significant protective effects against 5‐FU‐ and irinotecan‐induced mucositis in mice. FFTZS increased food intake, inhibited inflammatory infiltration in colonic tissues, and suppressed apoptosis induced by chemotherapy. By increasing goblet cell density, maintaining mucus layer integrity, and promoting sIgA secretion, FFTZS enhanced colonic immune function in chemotherapy‐treated mice. Furthermore, FFTZS elevated the expression of tight junction proteins Claudin‐1 and ZO‐1, improving histopathological damage. FFTZS also increased the relative abundance of beneficial bacteria such as *Roseburia*, *Peptococcus*, and *Odoribacter* while reducing the abundance of opportunistic pathogens in the colon. Additionally, FFTZS helped correct amino acid metabolic disturbances caused by 5‐FU and irinotecan treatment. Notably, our study underscores the potential clinical value of FFTZS in alleviating chemotherapy‐induced intestinal mucositis, highlighting the greater benefits of early intervention compared to late intervention. These findings hold important implications for developing adjunct therapies to mitigate the side effects of chemotherapy.

## Author Contributions

**Yongjun Kan:** data curation (equal), funding acquisition (equal), investigation (equal), methodology (equal), writing – original draft (equal). **Yingying Liu:** data curation (equal), funding acquisition (equal), investigation (equal), methodology (equal), writing – original draft (equal). **Li Zhao:** conceptualization (equal), investigation (equal). **Chang Jiang:** conceptualization (equal), investigation (equal). **Wensheng Pang:** conceptualization (equal), investigation (equal). **Bianhong Zhang:** visualization (equal). **Wenxiong Lin:** funding acquisition (equal). **Juan Hu:** data curation (equal), funding acquisition (equal), project administration (equal), writing – review and editing (equal).

## Conflicts of Interest

The authors declare no conflicts of interest.

## Supporting information


**Appendix S1:** fsn370789‐sup‐0001‐AppendixS1.docx.

## Data Availability

Data will be made available on request.
