# Supplementary material for: Fufang Taizishen Granules Attenuate Chemotherapy‐Induced Intestinal Mucositis by Modulating Gut Microbiota and Amino Acid Metabolism
Source: Food Sci Nutr. 2025 Aug 11;13(8):e70789. doi: 10.1002/fsn3.70789 (PMC12339415; doi:10.1002/fsn3.70789)
Supplement: Supplementary file 1 — Appendix S1: fsn370789‐sup‐0001‐AppendixS1.docx. [file FSN3-13-e70789-s001.docx]

**Fufang Taizishen Granules attenuate chemotherapy-induced intestinal mucositis by modulating gut microbiota and amino acid metabolism**

Yongjun Kan^a,1^, Yingying Liu^b,1^, Li Zhao^a^, Chang Jiang^a^, Wensheng Pang^c^, Bianhong Zhang^d^, Wenxiong Lin^d^ and Juan Hu ^a,b,*^

a *Fujian Academy of Chinese Medical Sciences, Fuzhou 350003, P.R. China*

b *The Second Affiliated Hospital of Fujian University of Traditional Chinese Medicine, Fuzhou, 350003, P.R. China*

c *Fujian University of Traditional Chinese Medicine, Fuzhou, 350122, P.R. China*

d *Fujian Key Laboratory for Agroecological Processes and Safety Monitoring, Fujian Agriculture and Forestry University, Fuzhou, 350002, P.R. China*

1 These authors contributed equally to this work and share first authorship.

* Correspondence: huj@fjtcm.edu.cn (J. Hu)

Permanent address： Fujian Academy of Chinese Medical Sciences, No.282 Wusi Rd, Gulou District, Fuzhou, Fujian, China.

**Section S1**

Table S1 Primer List for Akkermansia and Roseburia Detection by qPCR

| Name | Primer Type | Sequence | Ref |
| --- | --- | --- | --- |
| *Akkermansia* | Forward | CCTTGCGGTTGGCTTCAGAT | (Zhang et al., 2017) |
|  | Reverse | CAGCACGTGAAGGTGGGGAC |  |
| *Roseburia* | Forward | GCGGTRCGGCAAGTCTGA | (Seo et al., 2020) |
|  | Reverse | CCTCCGACACTCTAGTMCGAC |  |
| Bacterial | Forward | ACTCCTACGGGAGGCAGCAG | (Zhang et al., 2017) |
|  | Reverse | ATTACCGCGGCTGCTGG |  |

**References**

Seo, B., Jeon, K., Moon, S., Lee, K., Kim, W.-K., Jeong, H., . . . Ko, G. (2020). Roseburia spp. Abundance Associates with Alcohol Consumption in Humans and Its Administration Ameliorates Alcoholic Fatty Liver in Mice. *Cell Host & Microbe, 27*(1), 25-40.e26. doi:10.1016/j.chom.2019.11.001

Zhang, Z., Wu, X., Cao, S., Cromie, M., Shen, Y., Feng, Y., . . . Li, L. (2017). Chlorogenic Acid Ameliorates Experimental Colitis by Promoting Growth of Akkermansia in Mice. *Nutrients, 9*(7), 677. Retrieved from <http://dx.doi.org/10.3390/nu9070677> doi:10.3390/nu9070677


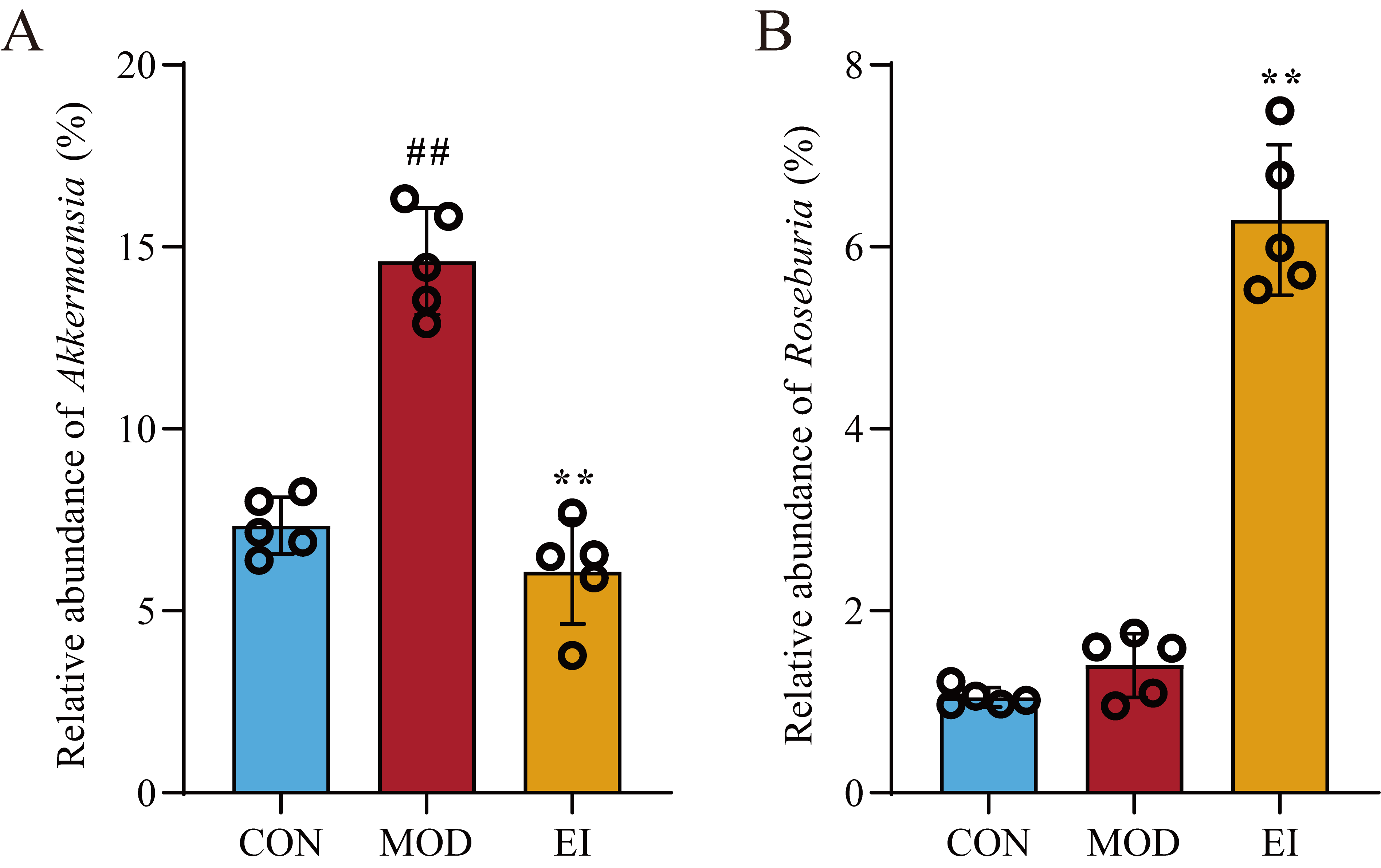


Fig. S1 Effects of FFTZS on the Relative Abundance of Akkermansia and Roseburia. (A) Comparison of the relative abundance of Akkermansia in fecal samples from mice treated with FFTZS. (B) Comparison of the relative abundance of Roseburia in fecal samples from mice treated with FFTZS. Data are presented as mean ± SD (n = 5). ##p < 0.01 vs. CON group; **p < 0.01 vs. MOD group.

**Section S2**

**(1) Chromatography conditions**

The column used was purchased from Waters Acquity UPLC HSS T3 column (1.8 μm 2.1 × 100 mm). A binary solvent system was employed, consisting of solvent A (water with 0.1% formic acid) and solvent B (acetonitrile with 0.1% formic acid). The elution was performed using the following gradient program: the run began with 98% A and 2% B, held for 0.25 min; solvent B was then linearly increased to 98% over 10 min and held until 13 min. At 13.1 min, the gradient was rapidly returned to initial conditions (98% A, 2% B), followed by re-equilibration until 15 min. The total run time was 15 minutes. The flow rate was set at 0.4 mL/min, and the injection volume was 1 μL.

**(2) Mass spectrum conditions**

Waters Xevo G2-XS QTOF high resolution mass spectrometer can collect primary and secondary mass spectrometry data in MSe mode under the control of the acquisition software (MassLynx V4.2, Waters). In each data acquisition cycle, dual-channel data acquisition can be performed on both low collision energy and high collision energy at the same time. The low collision energy is 2V, the high collision energy range is 10~40V, and the scanning frequency is 0.2 seconds for a mass spectrum. The parameters of the ESI ion source are as follows: Capillary voltage: 2000V (positive ion mode) or -1500V (negative ion mode); cone voltage: 30V; ion source temperature: 150°C; desolvent gas temperature 500°C; backflush gas flow rate: 50L/ h; Desolventizing gas flow rate: 800L/h. Mass-to-charge ratio (m/z) was acquired over a range of 50–1200.

**(3) Metabolite identification**

The raw data acquired using MassLynx V4.2 were processed with Progenesis QI software for peak extraction, alignment, and other data processing steps. Metabolite identification was performed based on the online METLIN database and an in-house Biomarker database within Progenesis QI, combined with theoretical fragment analysis. The mass tolerance was set within 100 ppm for precursor ions and within 50 ppm for fragment ions.
